# Diarrhea in suckling lambs is associated with changes in gut microbiota, serum immunological and biochemical parameters in an intensive production system

**DOI:** 10.3389/fmicb.2022.1020657

**Published:** 2022-11-17

**Authors:** Tao Zhong, Yaxuan Wang, Xinlu Wang, Aline Freitas-de-Melo, Hua Li, Siyuan Zhan, Linjie Wang, Jiaxue Cao, Dinghui Dai, Jiazhong Guo, Li Li, Hongping Zhang, Jinwang Liu, Lili Niu

**Affiliations:** ^1^Key Laboratory of Livestock and Poultry Multi-omics, Ministry of Agriculture and Rural Affairs, College of Animal Science and Technology, Sichuan Agricultural University, Chengdu, China; ^2^Departamento de Biociencias Veterinarias, Facultad de Veterinaria, Universidad de la República, Montevideo, Uruguay; ^3^Animal Nutrition Institute, Sichuan Agricultural University, Chengdu, China; ^4^Yulin Sannong Breeding Service Co., Ltd, Yulin, China

**Keywords:** *Ovis aries*, diarrhea, gut microbiota, 16S rRNA, immunology

## Abstract

The incidence of diarrhea in lambs is frequent in large-scale sheep farms, which greatly impacts the growth and health of lambs. The aim of this study was to assess the changes in serum biochemical and immunological parameters and gut microbiome composition in suckling lambs suffering from diarrhea or not, reared on an intensive commercial farm. We found a reduced diversity of intestinal bacteria in suckling lambs suffering from diarrhea. Firmicutes and Bacteroidetes were the dominant flora in both groups of lambs, while the Bacteroidetes decreased in diarrheic lambs, no changes were reported in Firmicutes. Compared with healthy lambs, the proportion of aerobic bacteria, facultative anaerobic bacteria, and stress tolerant bacteria increased in lambs suffering from diarrhea, while that of anaerobic bacteria and potentially pathogenic bacteria decreased slightly. In addition, the contents of total cholesterol, immunoglobulins (Ig) G, and IgM in the serum of lambs suffering from diarrhea were lower than those of healthy lambs. This study explored the association between diarrhea occurrence, intestinal microbial community structure, and metabolic and immunological status in Hu lambs.

## Introduction

In intensive sheep production systems, diarrhea is observed in lambs of all ages, but the highest incidence is observed between 22 to 35 days old, when diarrhea frequency can reach 25% (Wang et al., 2019). Diarrhea is associated with reduced body weight, growth retardation, and in severe cases, death, compromising farmers’ income (Aldomy and Zeid, 2007; Mariano et al., 2018). In lambs, diarrhea without other clinical signals is often associated with nutritional changes or imbalances, such as irregular feeding, inadequate quality and quantity of milk substitute, or maladaptation to concentrate rations (Sargison, 2004; Grosskopf et al., 2017; Wang et al., 2019). High densities, unhygienic conditions, and stressful situations (e.g., artificial weaning) give favor to the occurrence of diarrhea caused by bacteremia, parasitemia, and endotoxemia (Skirnisson and Hansson, 2006; Fortuoso et al., 2019). Infectious diarrhea affects a large proportion of the flock and is more commonly observed in housed lambs associated with other clinical signals (Sargison, 2004). From birth until 3 weeks old, the causes of diarrhea in lambs are associated with pathogenic infections or nutritional issues, such as low milk production by the mother, failure to suck, and maladaptation to food supplementation (Sobiech et al., 2013). Coccidiosis and helminthiasis are the most frequent pathogens involved in the incidence of diarrhea in suckling lambs from three to 12 weeks old (Sobiech et al., 2013). As diarrhea causes economic, health, and welfare concerns in growing lambs in intensive commercial farms, it is essential to generate healthy, robust, reliable biomarkers.

Diarrhea produces changes in total serum proteins (TP), glucose (Glu), blood urea nitrogen (BUN), and immunoglobulins G and M, as well as in the intestinal microbiome, which is required for the health development of the young (Sobiech et al., 2013; El-Deeb et al., 2020; Choi et al., 2021). In fact, several probiotics, such as *Streptococcus* and *Lactobacillus*, are more prominent in the feces of goat kids suffering from diarrhea than in healthy kids’ feces (Zhong et al., 2022). In mammals, including suckling ruminants, the gut is essential for digestion and absorption, colonized by a large number of microorganisms. These microorganisms maintain the steady-state phase of the gastrointestinal tract and play a crucial role in many physiological functions (Cummings and Macfarlane, 1997; Sonnenburg et al., 2004). The vast majority of studies have focused on the crosstalk between the gut microbiome changes and the host’s immune response to viral invasion or parasitic infection (Jasinska et al., 2020). However, there is a lack of studies on ruminants’ gut microbiome and diarrhea incidence in lambs, currently. The objective of this study was to assess the changes in biochemical and immunological parameters and gut microbiome composition in suckling lambs suffering or not, from diarrhea reared on an intensive commercial farm in China.

## Materials and methods

### Farm location, management, and sample processing

The experiment was conducted at an intensive commercial farm (Yulin Sannong Breeding Service Co., Ltd., Shaanxi, China) located at 38°38′ N and 109°87′ E with an average elevation of 1,224 m. This study used multiparous Hu ewes and their single lambs, homogeneous according to their lambing date. After lambing, the lambs were tagged, and their mothers and sex were identified. Lactating ewes and their lambs were housed together in sheepcotes in groups of 10 ewes and their lambs (2 m^2^ per animal). Corn silage and concentrate mixture were supplied twice daily at 9:00 h and 16:00 h (corn silage/concentrate ratio was 4:6; 1.5 to 2.0 kg/ewe), and water was provided in an automatic system. The ingredients and chemical composition of the concentrate mixture are presented in Supplementary Table S1. In addition, from 7 days old, an extra commercial starter concentrate was offered to the lambs through a creep feeding system until the end of the study. The ewes were treated with antiparasitic drugs before mating, and the lambs received vaccines for epidemic diseases (sheep pox, mycoplasma ovipneumoniae, peste des petits ruminants).

Fifty newborn lambs homogeneous according to body weight and sex (25 females, 2.50 ± 0.05 kg; 25 males: 2.78 ± 0.05 kg) were selected to record the incidence of diarrhea. From birth until 30 days of age, one veterinarian observed the occurrence of diarrhea daily, recording the lambs which presented past or liquid feces and fecal staining of the wool in the perineal region. Overall, 11 lambs were identified with diarrhea at 29 to 30 days of age (frequency of diarrhea: 22%; 11/50) (DL group; 4 females and 7 males), and other seventeen healthy lambs were selected randomly for this study (HL group; 9 females and 8 males). The HL lambs did not present diarrhea from birth until 30 days old. The DL lambs were sampled on the first day that presented diarrhea which lasted 2 or 3 days. No other clinical signals were found in the tested lambs during this experiment. The feces were collected with sterile swabs, which were inserted into the rectum of each lamb, and placed in 5-mL centrifugal tubes. Feces samples were stored at −80°C until further analysis. Thereafter, blood samples were collected from the jugular vein and stored in vacuum tubes without anticoagulants. Lambs were weighed 30 min after birth (BW) and at 30 days old.

### Parasites examination by microscopy

Microscopy of feces was performed to detect whether parasites were present in the experimental lambs using the flotation and sedimentation method (Khan et al., 2021). Approximately three grams of feces from each individual were mixed with a saturated salt solution and clarified with a filter. Then, saturated salt water was added to the filtered solution, and the eggs of parasites were observed floating in the liquid or not. Subsequently, eggs of parasites were detected by the smear examination. Lastly, the slides were photographed using a microscope (Olympus Corporation, Tokyo, Japan).

### Serum separation and biochemical assay

The blood samples were centrifuged immediately after collection at 3000 r/min for 10 min, and the serum was separated and stored at −20°C. An automatic biochemical analyzer (Model 7020, Hitachi, Tokyo, Japan) was used to determine the contents of IgG, IgM, total cholesterol (TC), total triglyceride (TG), Urea, total protein (TP), glucose (Glu), and albumin (Alb) according to a previous study (Zhong et al., 2022).

### DNA extraction and 16S rRNA sequencing

DNA was extracted from 28 stool samples using the TIANamp DNA stool Kit (Tiangen, Beijing, China) according to the manufacturer’s instructions. The concentration and purity of extracted DNA samples were measured using a NanoDrop 2000 spectrophotometer (Thermo Fisher Scientific, Waltham, MA, United States). The integrity of extracted DNA was determined by the 1% (w/v) agarose gel electrophoresis. Then, the DNA samples that passed quality inspection were amplified the V3-V4 hypervariable region of 16S rRNA by using the universal primers (338F and 806R) and sent to the Biomarker Biotechnologies Co., Ltd. (Beijing, China) for 16S rRNA sequencing using an Illumina sequencing platform (NovaSeq 6000, Illumina, San Diego, United States).

### Data analysis and statistical analysis

The Highseq platform was utilized to obtain the raw data and conduct quality control. The sequencing results were stored in FASTQ (FQ) format for subsequent analysis. The Usearch V10 software was applied to cluster the data with a similarity of 97.0% as the standard to form feature tables and feature sequences (Edgar, 2010). The representative sequences of each OTU were screened out for further annotation. In order to obtain taxonomic information and community composition, we annotated the feature sequences using the classifier and used SILVA as a reference database (Pruesse et al., 2007). QIIME2 software was used to analyze the Alpha and Beta diversities of the investigated samples, and the UPGMA hierarchical clustering tree was constructed (Caporaso et al., 2010). Four indexes, including Chao1, Shannon, Simpson, and ACE, were performed to estimate the alpha diversity among samples to explore their bacterial complexity. The NMDS results were used to illustrate the clustering. BugBase was used for functional annotation of the microbiome in lambs.

SPSS 27 was used for statistical analysis of the measured data. The normal distribution of all the variables was checked with the Shapiro–Wilk test. The data with normal distribution were compared by an independent T-test. Mann–Whitney test was used to compare the differences between two data sets that were not normally distributed. LEfSe was used to calculate and evaluate LDA (Linear Discriminant Analysis) to observe whether two groups of bacteria had significant changes. Analysis of similarity (ANOSIM) was performed to test the differences in bacterial community compositions across groups using weighted Unifrac. When *p*-value was less than 0.05, there was statistical significance.

## Results

### Effects of diarrhea on body weight, parasite eggs, biochemical and immunological profile

**Figure 1:**
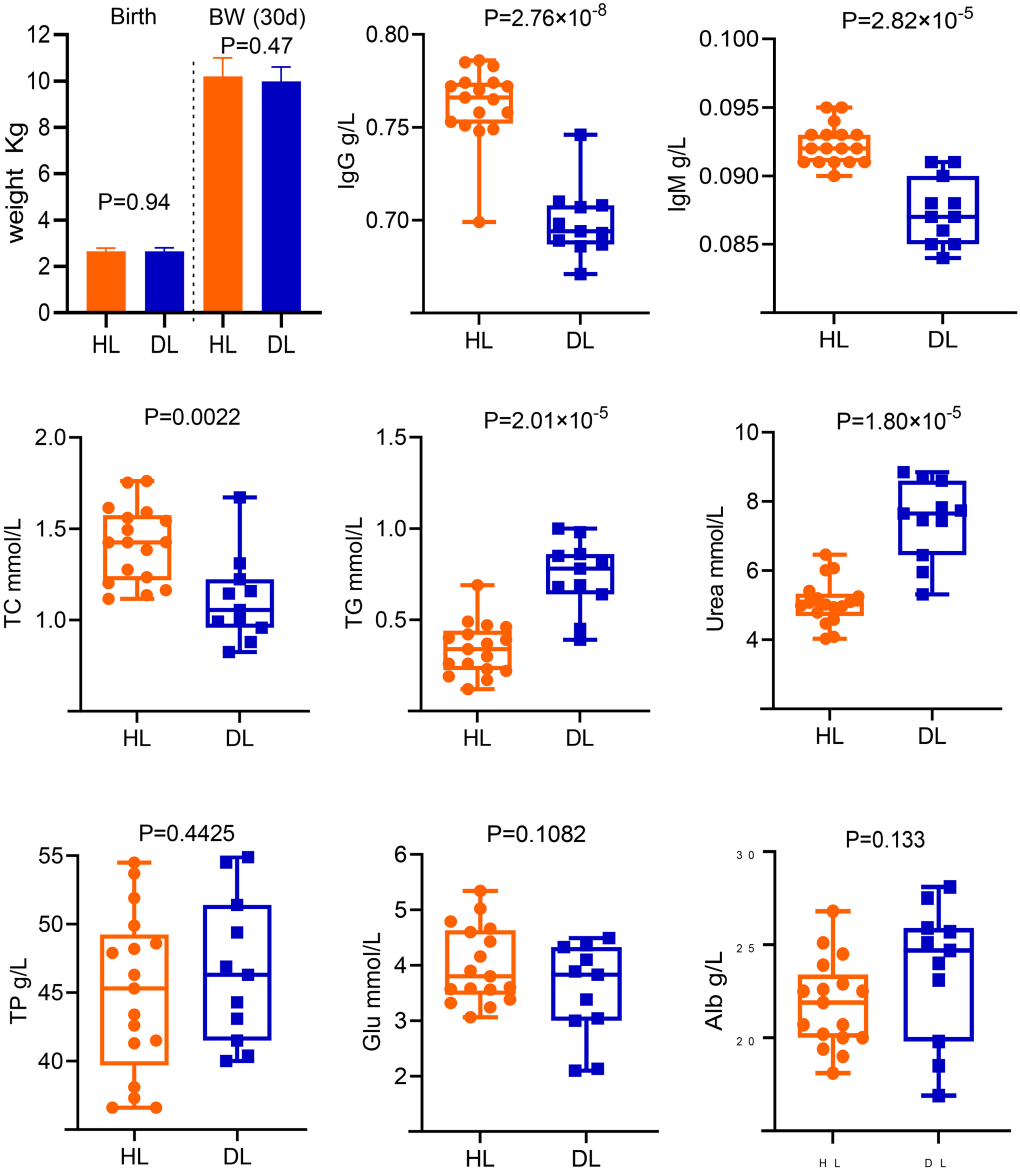
Determination of serum biochemical indices and immunological parameters of diarrheic and healthy lambs.

The body weight was not different between groups at birth and 30 days old (Figure 1). In addition, no parasite eggs were observed in both group (Supplementary Figure S1). However, there were significant differences in IgG, IgM, TC, TG, and Urea between DL and HL groups. The diarrheic lambs had lower concentrations of IgG, IgM, and TC (*p* < 0.01) than HL lambs. The concentrations of TG and Urea were higher in the DL group than those in the HL group (*p* < 0.001). Additionally, serum TP, Alb, Glb, and Glu concentrations did not differ between groups (Figure 1).

### Microbiota configuration in lambs suffering from diarrhea

**Figure 2:**
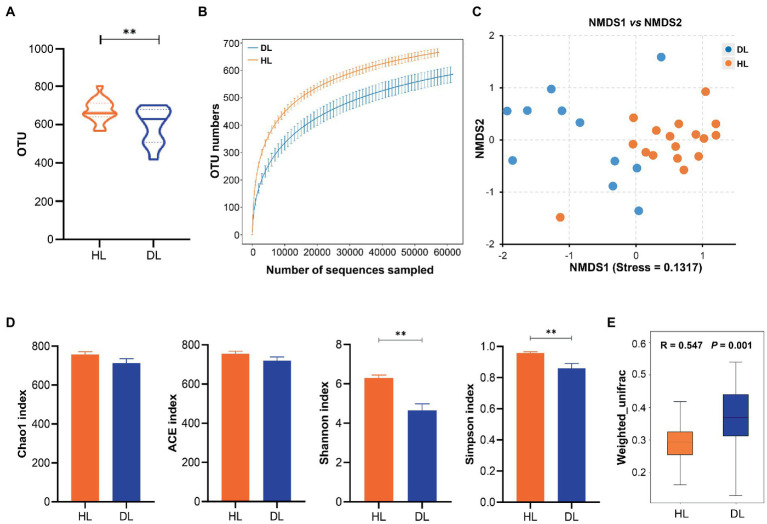
**(A)** The OTU numbers in each sample. **(B)** Multy sample Rarefaction curves. **(C)** NMDS analysis based in weighted Unifrac. **(D)** Alpha diversity index analysis. **(E)** Anosim analysis based in weighted Unifrac.

In total, a number of 2,103,398 effective sequences (raw sequences 2,223,345, effective rate 94.60%) and 18,008 operational taxonomic units (OTUs) were generated by similarity clustering (over 97%). The average Q30 value was 97.72 ± 0.059, which ensured the accuracy of sequencing (Table 1). The mean number of OTUs in the HL group was higher than that in the DL (Figure 2A). The rarefaction curve tended to be flat, indicating that the amount of sequencing data was progressive and reasonable (Figure 2B). Alpha diversity of the fecal bacterial community revealed that the Shannon and Simpson indices of the DL group were lower than those of the HL lambs (Figure 2D), which suggested that diarrhea could reduce microbial diversity and richness. The clustering of Non-metric Multidimensional Scaling (NMDS) showed that the HL and DL lambs were separated by the coordinate axis, and most of the HL lambs were located on the right of the graphic compared with the DL lambs (Figure 2C). Anosim reflected the beta diversity of samples. There were significant differences between the DL group and the HL group (*R* = 0.547, *p* = 0.001), and the difference between the two groups was greater than the difference within the group (Figure 2E). In addition, Principal-coordinate analysis (PCoA) illustrated that the healthy lambs harbored close weighted UniFrac distances (Supplementary Figure S2).

**Table 1:** OTU-based diversity indexes of gut microbiota in diarrheic and healthy lambs.

Sample	Raw reads	Clean reads	Effective (%)	Q20 (%)	Q30 (%)	GC (%)	OTUs	ACE	Chao1	Simpson	Shannon
DL01	75,384	75,384	94.20	99.54	97.60	53.33	680	797.9537	784.4393	0.7578	3.7393
DL02	79,396	79,396	87.22	99.59	97.78	53.05	508	646.1386	657.0000	0.9279	4.6269
DL03	79,377	79,377	95.05	99.60	97.86	52.97	674	763.1884	760.6849	0.9410	6.0374
DL04	79,365	79,365	93.93	99.58	97.79	53.96	630	741.3778	745.5542	0.7293	3.5166
DL05	79,643	79,643	97.71	99.56	97.69	52.53	702	765.9590	775.9859	0.9617	6.1520
DL06	79,628	79,628	96.60	99.59	97.82	53.20	680	747.2996	742.6582	0.9259	5.8860
DL07	79,635	79,635	96.32	99.55	97.64	53.15	636	768.5106	787.6395	0.8379	3.8053
DL08	79,677	79,677	92.39	99.57	97.71	51.90	587	747.6076	742.2500	0.9611	5.8350
DL09	79,529	79,529	94.55	99.56	97.69	54.48	494	585.8128	586.5000	0.6346	3.1804
DL10	79,128	79,128	89.09	99.55	97.61	52.63	419	674.0376	569.0476	0.8771	4.1836
DL11	79,524	79,524	91.50	99.57	97.73	50.42	522	670.9538	681.3889	0.8890	4.1163
HL01	79,727	79,727	95.14	99.56	97.71	53.55	657	725.2609	727.5455	0.9725	6.4926
HL02	79,542	79,542	95.90	99.56	97.68	52.62	694	781.3019	772.0645	0.9772	6.4126
HL03	79,546	79,546	96.43	99.58	97.78	53.46	688	745.1134	745.6761	0.9460	6.0051
HL04	79,774	79,774	96.70	99.57	97.78	53.92	737	843.2490	842.0333	0.8676	5.0179
HL05	79,558	79,558	96.68	99.57	97.74	52.87	708	770.5018	763.5584	0.9490	6.3254
HL06	79,547	79,547	91.14	99.56	97.69	52.14	718	793.8739	804.9531	0.9776	6.8963
HL07	79,579	79,579	93.19	99.58	97.74	51.80	741	819.4298	820.7841	0.9142	5.4631
HL08	79,462	79,462	96.28	99.56	97.70	52.48	803	855.6519	874.6557	0.9712	6.8790
HL09	79,893	79,893	94.68	99.57	97.72	52.42	652	736.3111	729.6849	0.9757	6.6244
HL10	79,211	79,211	97.19	99.57	97.73	53.52	656	729.9679	740.6418	0.9604	6.1588
HL11	79,317	79,317	94.26	99.57	97.72	52.56	705	760.1582	761.7167	0.9818	7.0873
HL12	79,564	79,564	95.71	99.57	97.75	54.05	569	649.2778	657.3929	0.9739	6.3689
HL13	79,752	79,752	96.87	99.55	97.67	53.32	618	681.7643	693.0588	0.9838	7.0329
HL14	79,262	79,262	95.84	99.57	97.70	53.40	632	710.0753	707.0429	0.9799	6.7342
HL15	79,514	79,514	93.51	99.55	97.63	51.85	579	701.6997	690.8000	0.9555	5.7329
HL16	79,738	79,738	96.12	99.57	97.76	53.12	661	764.6017	784.4058	0.9335	5.6272
HL17	80,073	80,073	94.66	99.55	97.68	52.52	658	760.3122	768.5844	0.9677	6.2275

**Figure 3:**
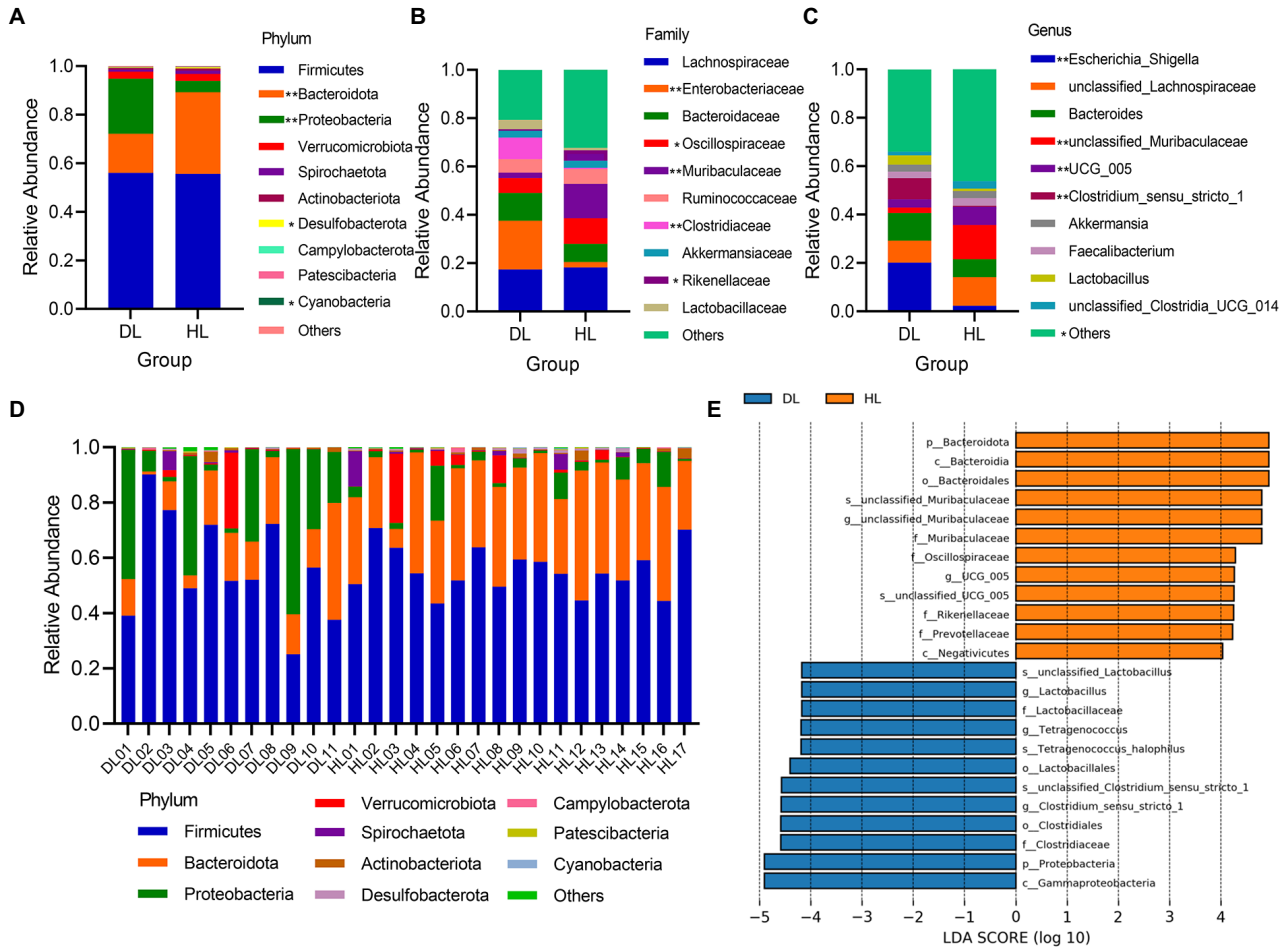
**(A–C)** The relative abundance of the top 10 between DL and HL groups of phylum, families, and genus, respectively. **(D)** The relative abundance of the top 10 phyla in every individual. **(E)** LDA histogram of species differences between diarrheic and healthy lamb groups.

At the phylum level, Firmicutes (DL = 56.02%; HL = 55.64%) and Bacteroidetes (DL = 16.08%; HL = 33.59%) were the dominant bacterial components. The relative abundance of Firmicutes was not different between groups, but Bacteroidetes (*p* < 0.001), with significant differences, were greater in HL than in DL lambs (Figure 3A). The relative expression of bacteria in DL lambs was higher than HL group. Firmicutes, Bacteroidetes, and Proteobacteria were the dominant flora in most samples. Especially in DL02, Firmicutes accounted for more than 80%, while in DL09, Proteobacteria became the dominant bacterial community (Figure 3D). This suggests that different individuals with the same health status also have differences in microbial community structure. In addition, Desulfobacterota (*p* = 0.0160) and Cyanobacteria (*p* = 0.0499) were significant between DL and HL groups (Figure 3D). At the family level, except for the other unknown bacterial groups, *Lachnospiraceae* was the most abundant. Moreover, relative abundance of *Enterobacteriaceae (p =* 0.0039*)*, *Oscillospiraceae* (*p* = 0.0181), *Muribaculaceae* (*p* = 0.0078), *Clostridiaceae* (*p* = 0.0053) and *Rikenellaceae* (*p* = 0.0197) were significantly different between groups (Figure 3B). In the genus, *Escherichia_shigella* (*p* = 0.0039), *unclassified_Muribaculaceae* (*p* = 0.0078), *Clostridium_sensu_stricto_1* (*p* = 0.0053), *UCG_005* (*p* < 0.001) and the other flora (*p* = 0.0179) were significantly different between groups (Figure 3C). LEfSe analysis showed that the main enrichments of fecal bacteria in the HL lambs were Bacteroidetes*, Muribaculaceae,* and *Oscillospiraceae*, and in DL lambs, were mainly *Lactobacillus, Clostridia, Tetragenococcus*, and other species (Figure 3E).

### Functional consequences in microbial communities induced by diarrhea

**Figure 4:**
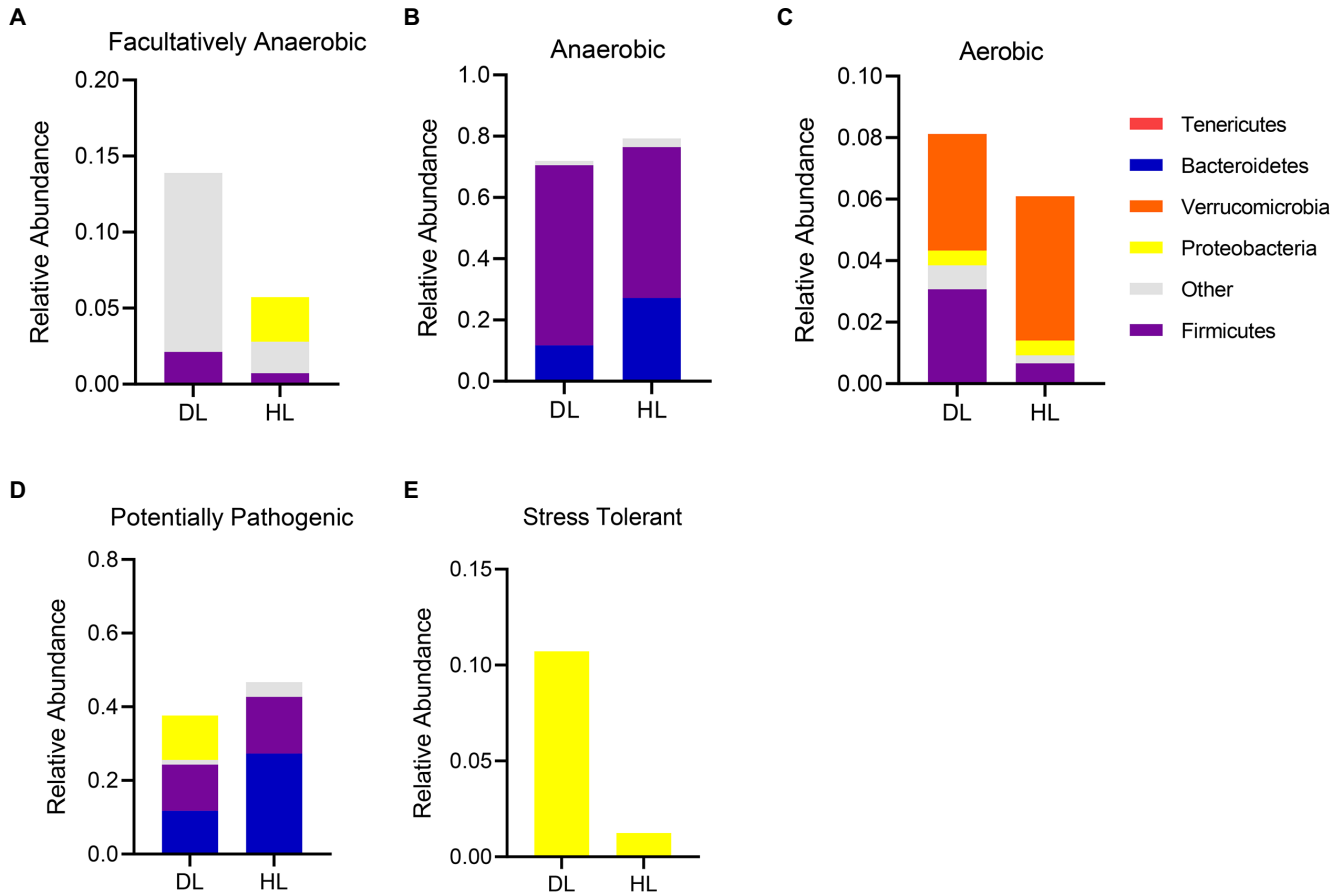
BugBase functional analysis of intestinal microbiota in each group. **(A)** Facultative anaerobe compositions in each group. **(B)** Anaerobic bacteria compositions in each group. **(C)** Composition of aerobic bacteria in each group. **(D)** Composition of Potentially Pathogenic in DL and HL group. **(E)** Composition of Stress Tolerant in each group.

The bacterial community functions were annotated by BugBase, and the proportion trend of bacteria at the phylum level was observed between the two groups so as to judge the relationship between the occurrence of diarrhea and the bacterial community functions. The BugBase analysis showed the relative abundance distribution of phylum in both groups of lambs (Figure 4). The composition of facultative anaerobic bacteria was different between groups. The HL group was mainly derived from Tenericutes and Proteobacteria, while the DL group was derived from Proteobacteria and other bacteria (Figure 4A). It is worth mentioning that the proportion of aerobic bacteria and facultative anaerobic bacteria increased in DL lambs, but the proportion of anaerobic bacteria decreased slightly in this group. The sources of anaerobic bacteria did not differ between groups, but Firmicutes were more abundant in DL than in HL lambs (Figures 4B,C). In addition, the content of stress-tolerant bacteria greatly increased in the DL group. The content of potentially pathogenic bacteria decreased in DL compared with the HL group, while stress tolerant bacteria were more abundant in the DL group (Figures 4D,E). Some potentially pathogenic bacteria from the Proteobacteria phylum were found in DL lambs.

## Discussion

The incidence of diarrhea affected the serum biochemical, immunological status, and gut microbiome composition of suckling lambs reared in an intensive production system. These differences were observed in suckling lambs at 29–30 days old, ages considered within the range of the highest occurrence of diarrhea in intensive production systems (Wang et al., 2019). The intestinal bacterial community composition differs at phylum, family, and genus levels between DL and HL groups. The number of OTUs and the Chao1, Shannon, Simpson, ACE index was lower in DL lambs than in HL lambs, indicating that diarrhea reduced the abundance of bacteria. Our results coincide with the results from studies performed in pigs, which found that the abundance and diversity of intestinal microbiota were reduced due to diarrhea (Pop et al., 2014; Hermann et al., 2015). We found that Firmicutes and Bacteroidetes were the dominant bacteria in both groups of lambs, as previously reported in suckling lambs (Looft et al., 2014; Bi et al., 2019). In addition, the proportion of Bacteroidetes in lambs with diarrhea decreased, while the content of Proteobacteria increased. It has been reported that Bacteroidetes were closely related to weight loss in humans (Ley et al., 2006), while Proteobacteria included many pathogens that were closely related to the diagnosis of intestinal diseases (Evans et al., 2011). *Cyanobacteria* have the functions of vitamin B and K synthesis, obligate anaerobic fermentation, and so on. It has also been found *Cyanobacteria* are one of the main phyla of intestinal microorganisms in sheep (Di Rienzi et al., 2013; Zhang et al., 2018). The occurrence of diarrhea was related to the decrease of *Cyanobacteria* content, and the digestion of lambs may be affected to some extent. The content of *Enterobacteriaceae* in lambs with diarrhea was much higher than that in the healthy group. *Enterobacteriaceae* includes *Shigella*, *Salmonella,* and other bacteria that affect the intestinal health of lambs, and enterotoxigenic *Escherichia coli* (ETEC) was also the main cause of diarrhea in livestock (Dubreuil et al., 2016). At the genus level, the relative content of *Escherichia_shigella* in DL lambs increased significantly. *Escherichia_shigella* is a conditionally pathogenic bacteria (Kong et al., 2019). *Oscillospiraceae* is one of the important components of feces, which can produce butyrate and is highly related to animal health (Konikoff and Gophna, 2016). Diarrhea also caused an increase of *Clostridium* content in the gut of suckling lambs, which might benefit nutrient digestion and absorption, as it is closely associated with the maintenance of intestinal microbiome balance (Louis and Flint, 2009; Atarashi et al., 2011). Intestinal infection caused by *Clostridiaceae* was relatively common, which also explained the significant difference in the content of this genus between the diarrheic and the healthy group (Uzal et al., 2018). The abundance of *Lactobacillus* in the intestinal tract of the DL group was higher than that in the HL group. The breast milk replacer likely provided adequate conditions in the gut for lactic acid bacteria to grow, increasing the abundance of Lactobacillus. In a previous study of our research group, we also found that Streptococcus and Lactobacillus were more prominent in the feces of weaned goats that received milk substitutes and suffered from diarrhea (Zhong et al., 2022). In this sense, the consumption of unpasteurized milk is associated with increased lactobacillus abundance in the human gut microbiome (Butler et al., 2020). We speculate that *Lactobacillus* avoided the worst health status in the DL group as it prevents pathogens from overgrowing in the intestine (Charlet et al., 2020). The higher content of *Tetragenococcus* in the DL group may be related to the function of regulating the body’s immunity and alleviating the occurrence of worse conditions such as weight loss and enteritis (Islam et al., 2022).

In addition, we found a significant reduction in the relative content of *unclassified_muribaculaceae* bacteria in the DL group. The abundance of feces of *Muribaculaceae* is strongly associated with longevity in Spalax Leucodon (Sibai et al., 2020). Other studies have also shown that *Muribaculaceae* is beneficial to lipid metabolism (Zhang et al., 2021), which might explain the lower concentration of TC in DL lambs than in HL lambs. We found that Firmicutes, Bacteroidetes, and Proteobacteria were the prominent bacteria of intestinal microflora in lambs, in agreement with studies in goat kids and steers (de Oliveira et al., 2013; Zhong et al., 2022). Firmicutes are closely related to energy acquisition and immune response regulation of the body (Zhang et al., 2015), while Bacteroidetes are one of the anaerobic bacteria in the intestinal tract related to polysaccharide absorption (Bjursell et al., 2006; Le Huerou-Luron et al., 2010). Proteobacteria include many pathogenic bacteria, which can cause diarrhea and other intestinal diseases (Grotiuz et al., 2006). Among the potentially pathogenic bacteria, *Enterobacteria*, *Bacteroides,* and *Faecalibacterium* were the main components of Proteobacteria. *Enterobacteria* was the only main bacteria of Proteobacteria in stress tolerant bacteria (Figures 4D,E). The related potential pathogenic flora has also been studied in piglets with diarrhea (Jonach et al., 2014; Malik et al., 2017). While most research on stress tolerant flora has been done in plants (Verma et al., 2016; Saijo and Loo, 2020), we speculate that Enterobacterium may have functions related to stress tolerance.

Serum biochemical parameters reflect the metabolic and health statuses of the animal body. Some biochemical parameters that indicate metabolic status differed between groups (TC, TG, and Urea). In this sense, as the body weight did not differ between groups, we speculate that nutrition was not the main influencing factor. A positive correlation has been found between serum IgG concentration and health status in calves, which is consistent with the results of this experiment (Furman-Fratczak et al., 2011). Diarrheic lambs had lower concentrations of TC and greater concentrations of TG (Geng et al., 2021), indicating that diarrhea could affect the efficiency of lipid metabolism. Salem et al. (2018) found that TG and Urea in the serum of diarrheic puppies were higher than those of healthy puppies, which is consistent with our results. However, different animal breeds, ages, and diarrhea etiology perhaps being the cause. The serum IgG and IgM concentrations of DL lambs were significantly lower than those of HL lambs, suggesting that the immune level of DL lambs decreased. Overall, even though our experimental design did not allow us to study the cause-effect relationship between the presence of diarrhea and changes in the studied parameters, individual differences in metabolism and immunological status likely led to diarrhea. All the lambs received similar nutritional and health management and were housed in the same surrounding. Therefore, these factors probably were not the causes of diarrhea.

Our study analyzed the serum biochemical and immunological indices and fecal microbial community composition of one-month-old lambs suffering from diarrhea or not, during the preweaning period. The diversity and richness of bacteria in the feces of HL lambs were higher than in DL lambs, likely benefiting the healthy growth of lambs. Diarrheic lambs had a poorer immunological status than HL lambs. Serum biochemical parameters in lambs suffering from diarrhea indicate differences in lipid and protein metabolism. This study explored the association between intestinal microbial community structure, biochemical and immunological statuses, and diarrhea in suckling lambs and provided a theoretical reference point for the treatment of diarrhea in lambs. These results highlight the need for further studies to determine the cause-effect relationship between the presence of diarrhea and changes in biochemical and immunological parameters and gut microbiome composition in lambs reared in intensive sheep production systems.

## Data availability statement

The 16s rRNA sequencing data were submitted to NCBI with the accession No. PRJNA853133.

## Ethics statement

The animal study was reviewed and approved by Institutional Animal Care and Use Committee at the College of Animal Science and Technology, Sichuan Agricultural University.

## Author contributions

TZ, JL, and LN conceived and designed the experiments. XW and JL collected relative samples. YW, XW, and HL performed the experiments. YW, JC, LW, SZ, LL, and HZ participated in data analysis. DD, JG, and TZ contributed reagents and materials. YW, AF-d-M, and TZ wrote and revised the manuscript. All authors have read and approved the final manuscript.

## Funding

This research was supported by the Open Fund of Farm Animal Genetic Resources Exploration and Innovation Key Laboratory of Sichuan Province, grant number cndky-2021-02 and the Scientific Research Interest Training Program of Sichuan Agricultural University (2022373).

## Conflict of interest

JL was employed by Yulin Sannong Breeding Service Co., Ltd.

The remaining authors declare that the research was conducted in the absence of any commercial or financial relationships that could be construed as a potential conflict of interest.

## Publisher’s note

All claims expressed in this article are solely those of the authors and do not necessarily represent those of their affiliated organizations, or those of the publisher, the editors and the reviewers. Any product that may be evaluated in this article, or claim that may be made by its manufacturer, is not guaranteed or endorsed by the publisher.
