# Supplementary figures and images for: Diarrhea in suckling lambs is associated with changes in gut microbiota, serum immunological and biochemical parameters in an intensive production system
Source: Front Microbiol. 2022 Nov 17;13:1020657. doi: 10.3389/fmicb.2022.1020657 (PMC9712182; doi:10.3389/fmicb.2022.1020657)

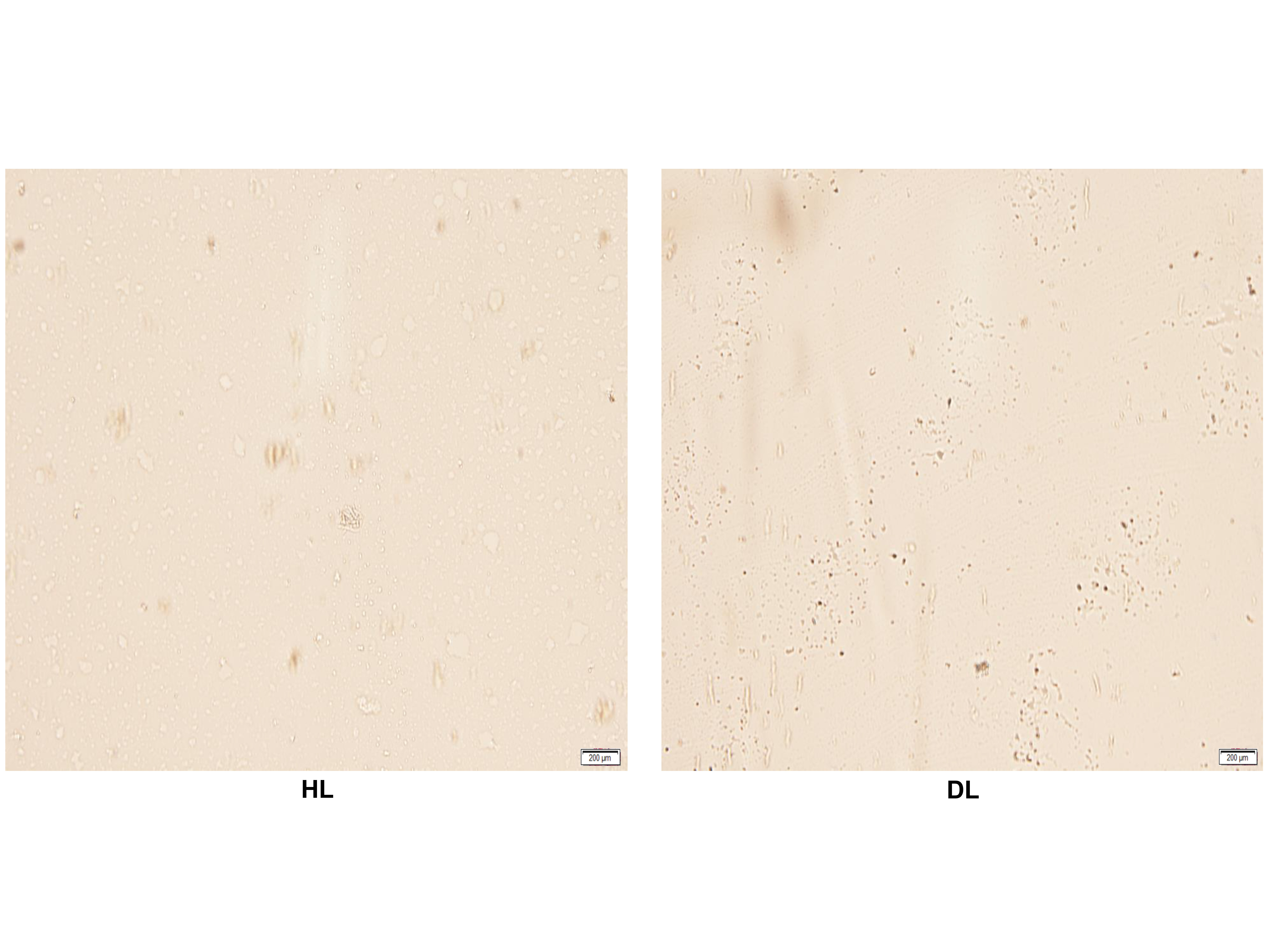

Supplement: Supplementary file 1 [file Image_1.JPEG]

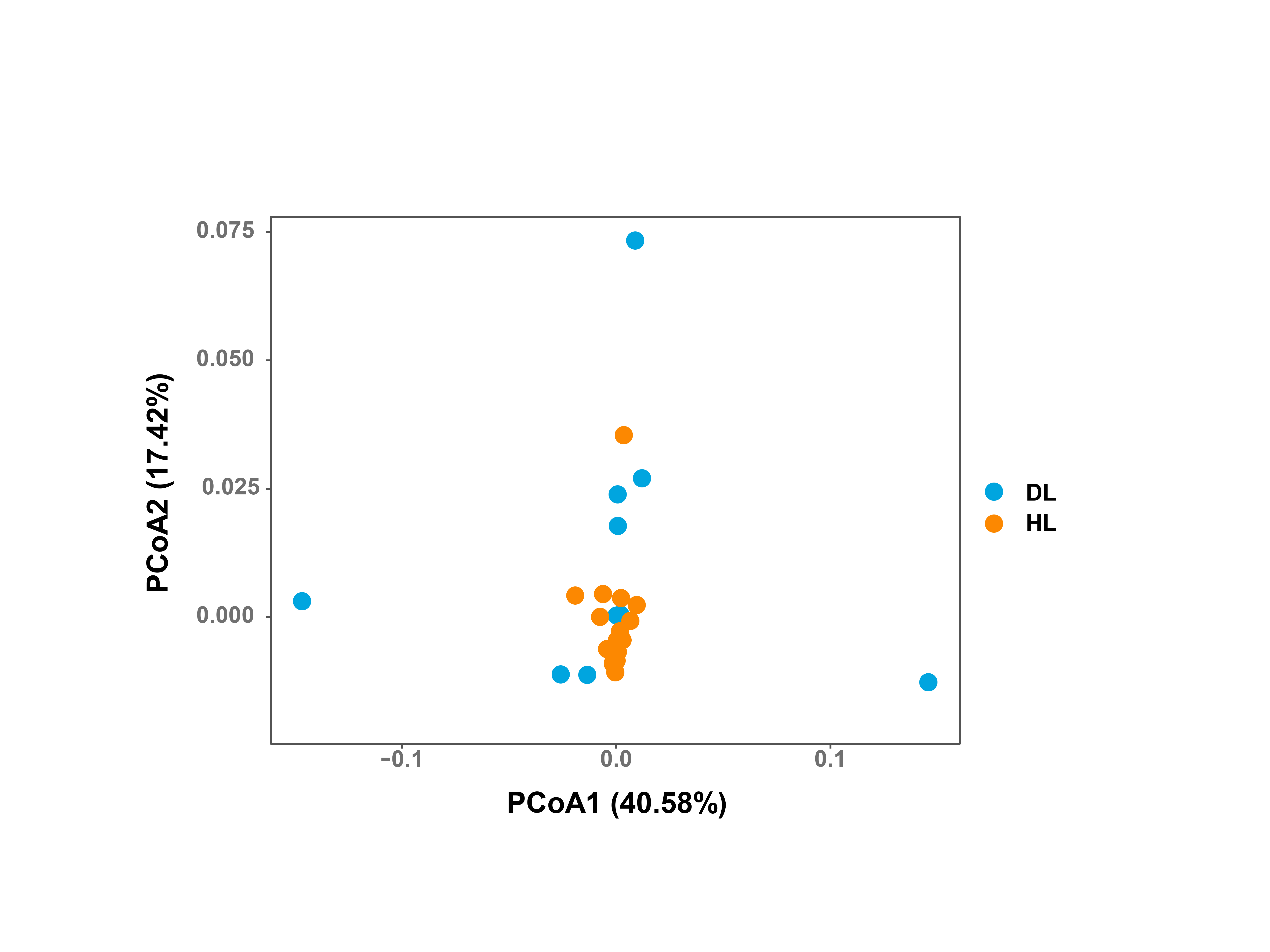

Supplement: Supplementary file 2 [file Image_2.JPEG]
